# Impact of aerobic and resistance training on fatigue, quality of life, and physical activity in prostate cancer patients: a systematic review and meta-analysis

**DOI:** 10.1097/JS9.0000000000000982

**Published:** 2024-01-15

**Authors:** Simon Nader, Amr Massoud, Feras Al-Obeidat, Waleed F. Mohamed, Wael Hafez, Asrar Rashid, Omar A. E. Yousef, Muneir Gador, Sabah Ahmed, Mohan Jose, Ahmed Abdelrahman, Mahmoud abdelshakour, Sherihan Fathey, María F. Osorio, Karla Robles-Velasco, Iván Cherrez-Ojeda

**Affiliations:** a NMC Royal Hospital Royal Hospital, Abu Dhabi, United Arab Emirates; b College of Technological Innovations, Zayed University, AbU Dhabi, United Arab Emirates; c Healthpoint Hospital, Abu Dhabi, United Arab Emirates; d Internal Medicine Department, Medical Research and Clinical Studies Institute; The National Research Center, 33 El Buhouth St, Ad Doqi, Dokki, Cairo Governorate, Cairo, Egypt; e Department of health, Giza, Egypt; f Faculty of Medicine, University of Debrecan, Debrecen, Hungary; g Department of Allergy, Immunology and Pulmonary Medicine, Universidad Espíritu Santo, Samborondón, Ecuador, & Respiralab Research Group, Guayaquil, Ecuador

**Keywords:** cancer rehabilitation, exercise therapy, oncology, physical endurance, quality of life, resistance training

## Abstract

**Background:**

Prostate cancer (PCa) is a prevalent cancer with significant morbidity and mortality rates. In most cases, PCa remains asymptomatic until advanced disease manifests with symptoms, such as benign prostate hyperplasia. Timely detection and better management have improved overall survival in patients with PCa, and fatigue, reduced physical activity, and impaired quality of life (QoL) remain major challenges that impact daily life.

**Objective:**

This study aimed to systematically review and conduct a meta-analysis to evaluate the impact of aerobic and resistance training on fatigue, QoL, and physical activity in PCa patients undergoing treatment.

**Material and methods:**

A comprehensive literature search was conducted using the PubMed, Cochrane Library, and clinicaltrials.gov databases, adhering to the PRISMA guidelines. Twenty studies, involving 1393 participants, were included in the final analysis. The inclusion criteria were studies that evaluated the effects of exercise interventions relative to passive controls in patients with PCa were included. The primary outcomes of interest were fatigue, QoL, and PA. Data from eligible studies were extracted, and a meta-analysis was performed using RevMan 5.40.

**Results:**

Twenty studies met our inclusion criteria. Data analysis of the included studies demonstrated a significant improvement in QoL among PCa patients in the exercise group compared to the control group (SMD=0.20, 95% CI=0.07–0.34, *P*=0.003). However, there was no significant association between exercise and fatigue (SMD=0.07, 95% CI=−0.13–0.26, *P*=0.51). Sensitivity analysis did not alter these findings. Regarding physical activity outcomes, the control group exhibited superior performance in the 400 m walk test (*P*<0.05). No significant associations were found between exercise and the 6 m walk test or up-and-go time.

**Conclusion:**

This systematic review revealed that aerobic and resistance training enhance the QoL of patients with PCa, although it has a limited impact on fatigue and physical activity levels. These findings advocate a shift in clinical practice and the positioning of exercise as a core component of comprehensive cancer care. Tailoring exercise regimens according to individual patient needs and treatment stages should become the norm in treatment planning. This approach goes beyond physical wellness and addresses the psychological and emotional facets of cancer management. Moreover, there is an evident need for further research to develop holistic exercise interventions that effectively address the complex dynamics of fatigue, physical activity, and QoL in this patient group.

## Introduction

Highlights

- Prostate cancer (PCa) patients struggle with fatigue, limited activity, and a low quality of life (QoL).
- This systematic review investigates how resistance and aerobic exercise impact fatigue, QoL, and physical activity in PCa patients, presenting a comprehensive analysis of 20 studies.
- Exercise improved PCa patients’ QoL compared to the control group.
- There was a lack of association between exercise and fatigue, the 6 m walk test, or up-and-go time.
- Our analysis encourages further research exploring additional strategies to address fatigue and promote physical activity in PCa demographics.

Globally, prostate cancer (PCa) is the second most common cancer in males and the fourth most common cancer overall, with over 1.4 million new cases reported in 2020. While it remained more prevalent in high-income countries such as France, Ireland, and Sweden, Zimbabwe reported the highest death count in 2020^1^. Although the precise pathogenesis of PCa is complex, several factors are known to contribute, including increasing age, ethnicity, obesity, increased height, hypertension, sedentary lifestyle, smoking, and chronically elevated testosterone levels. Similarly, several genetic mutations have been linked to it, including those in hereditary prostate cancer (HPC) gene 1, HPC 2, CAPB, and TMPRSS2-ETS gene families^2^.

In most cases, PCa remains asymptomatic until advanced disease manifests with symptoms, such as benign prostate hyperplasia. The disease may present with urinary urgency, frequency, difficulty in voiding, compromised renal function, impotence, or features of metastatic disease involving the bones, lymph nodes, rectum, and nervous system. Digital rectal examination (DRE) is an integral part of PCa screening; however, its positive predictive value is lower than that of prostate-specific antigen (PSA). However, DRE may have a lower positive predictive value and still has significant clinical value, especially in identifying advanced-stage diseases. DRE can be effective for detecting PCa that has progressed to a more advanced and palpable stage. Serum PSA level plays a pivotal role in determining high-risk prostate adenocarcinoma patients, with a positive predictor potential depending on the cut-off values. In patients with clinical suspicion, a transrectal ultrasound-guided needle biopsy sample was obtained to reach a definitive histological diagnosis. Similarly, the Prostate health index, which statistically combines tPSA, fPSA, and [-2] pro-PSA to offer better specificity and sensitivity, can also be employed^3^.

Following diagnosis, PCa was classified using the American Joint Committee on Cancer Tumor Node Metastasis (AJCC TNM) staging system. Staging uses the extent of the primary tumor (T), spread to lymph nodes (N), metastasis (M), PSA levels, and Gleason score grade group to categorize tumors into stages I, IIa, IIb, IIc, IIIa, IIIb, IIIc, IVa, and IVb^4^.

Although several management options are available, radical prostatectomy, with or without radiotherapy, remains the mainstay of treatment for localized diseases. This may be accompanied by lymph node removal, depending on sentinel lymph node involvement. Additionally, external beam radiotherapy can be used independently to manage low-risk, intermediate-risk, and high-risk patients following prostatectomy. Brachytherapy offers the benefit of delivering tumor-focused radiation therapy while sparing the surrounding tissues and can be either used alone or in conjugation with external beam therapy^5^. Androgen deprivation therapy impairs testosterone and other male hormone production, and prevents the stimulation of tumor growth. This can be accomplished via bilateral orchiectomy or medical castration induced by a series of drugs such as flutamide and chlormadinone acetate. Finally, chemotherapy with agents such as Docetaxel and Cabazitaxel can also be used in cases of advanced disease^6^.

Timely detection and better management have improved overall survival in patients with PCa; fatigue, reduced physical activity, and impaired quality of life (QoL) remain major challenges that impact daily life^7^. According to one study, 78% of patients seeking treatment for PCa reported significant fatigue that impacted their routine life activities^8^. In this systematic review and meta-analysis, we evaluated the impact of aerobic and resistance training relative to usual care on fatigue, QoL, and physical activity in patients with PCa undergoing treatment.

The American College of Sports Medicine (ACSM) defines aerobic exercise as an activity that utilizes large muscle groups while maintaining them and is performed rhythmically. These exercises use aerobic metabolism for energy generation. Aerobic exercises include cycling, running, swimming, etc.^9^. These exercises improve cardiovascular fitness and enhance the ability of the body to use oxygen efficiently. Resistance training is a form of periodic physical activity that involves the use of external weights or resistance against force to provide muscles with a progressive overload. It is a form of anaerobic exercise^10^.

Thus, this systematic review aimed to evaluate the effects of aerobic and resistance training on fatigue, QoL, and physical activity in PCa patients, providing a thorough synthesis of the current research findings.

## Material and methods

This systematic review and meta-analysis was conducted in accordance with the Preferred Reporting Items for Systematic Review and Meta-analysis (PRISMA)^11^ and Assessing the Methodological Quality of Systematic Reviews (AMSTAR) guidelines^12^, and the protocol was registered in PROSPERO

### Data source and search strategy

The authors conducted a thorough literature search from inception till 30th May 2023, using PubMed, the Cochrane Library, and clinicaltrials.gov. To obtain comprehensive search results, similar terms, synonyms, and spelling variants were employed. Our search string is comprised the following terms: physical activity, exercise, aerobic exercise, resistance training, physical inactivity, prostate carcinoma, prostate neoplasm, and PCa. Following a preliminary search, duplicates were removed using Zotero, a manual duplicate removal tool, and the recruited articles were screened for their full length.

### Inclusion and exclusion criteria

Studies were included only if they evaluated the impact of aerobic and/or resistance training relative to passive control on fatigue, QoL, and physical activity among patients undergoing PCa treatment. We did not limit the studies to the presence or absence of metastasis or the effects evaluated in patients receiving any particular management. We included only original studies, such as randomized controlled trials, cohort observational studies, and case–control studies.

Studies were excluded if they evaluated the impact of combined interventions such as exercise and medicine, if an active control was used, or if they studied the impact on nondesired outcomes such as inflammatory biomarkers. Similarly, articles that employed physical interventions other than aerobic and resistance training were deemed ineligible for inclusion. Finally, all case reports, single-arm studies, reviews, and meta-analyses were excluded.

### Data extraction and quality assessment

Following a comprehensive literature search, relevant information from the recruited articles was tabulated using an Excel spreadsheet. The following measurements were recorded: author’s name, study year, number of participants, intervention, control, intervention exercise details, and outcomes.

To assess the quality of included trials, the Cochrane Risk of Bias tool (The Nordic Cochrane Centre, Copenhagen, The Cochrane Collaboration) was used, which categorizes each study as either high-risk, low-risk, or unclear across the following domains: random sequence generation, allocation concealment, selective reporting, blinding of participants/personnel, blinding of outcome assessment, incomplete outcome data, and any other source of bias.

### Outcomes

This systematic review and meta-analysis aimed to evaluate the impact of aerobic or resistance training relative to control in patients with prostate carcinoma. The primary outcomes of interest were fatigue, QoL, and PA. We used the 400 m walk, 6 m walk, and up-and-go tests to assess physical activity levels. Furthermore, these studies were not limited to the scales and tools employed to assess the desired outcomes.

### Statistical analysis

To determine the pooled effects, Review Manager (RevMan) 5.40 (The Nordic Cochrane Centre, Copenhagen, The Cochrane Collaboration, 2014) was used. Given the variations in exercise programs and tools employed to assess outcomes, a random-effects model was used to determine the standard mean difference, confidence interval, and *P*-value. Heterogeneity was assessed using I2, and a value greater than 75% was considered significant.

## Results

### Literature search

**Figure 1:**
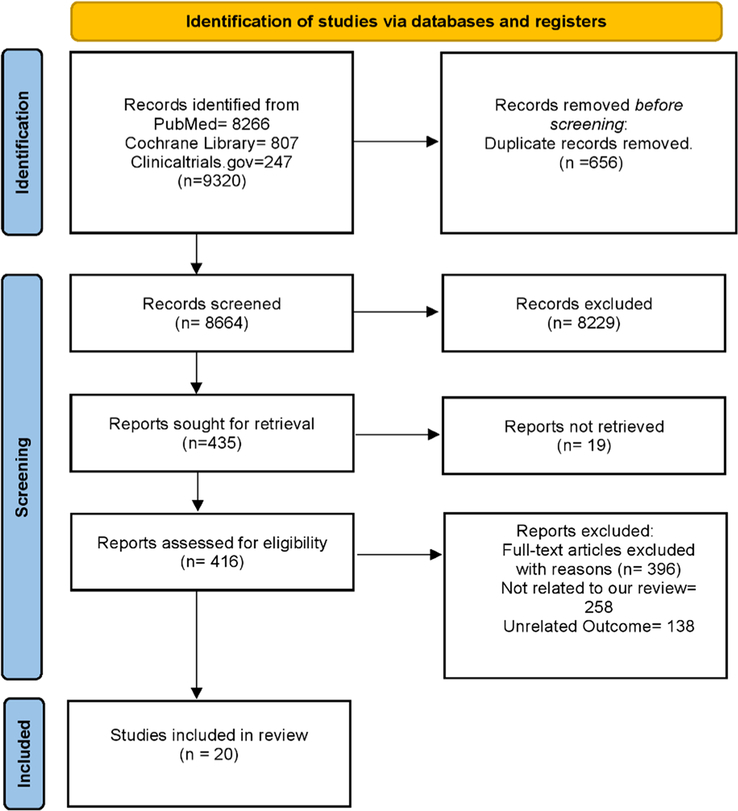
PRISMA Flow Chart.

Our initial electronic database search yielded 9320 articles. After removing duplicates and screening by title and abstract, 435 articles were excluded, of which 19 articles could not be retrieved. Finally, 416 articles were considered eligible for full-length review. Following a thorough assessment of these articles, 20 were ultimately considered eligible for inclusion in this systematic review and meta-analysis^7,13–31^. All studies included in this meta-analysis met our predefined inclusion criteria, as described in the Materials and Methods section. The results of our literature review are summarized in the PRISMA flowchart (Fig. 1).

### Study characteristics

A total of 1393 patients (*I*=731, *C*=662 ) were included in our study. The mean age for the intervention and control groups were 69.42±7.6 and 68.98±8.05, respectively, while the BMI was 28.13±4.1 kg/m² for the intervention while 27.48±3.43 kg/m² for the control group. Four of our studies employed aerobic training, five had resistance, while 10 had both aerobic and resistance training. Only one study had high load strength as its training component. The details of the included studies’ characteristics are tabulated in Table 1, and the outcomes are presented in Table 2.

**Table 1:** Study characteristics.

				Population characteristics		
Author (year)	Study type	interventions	Intervention details	Intervention	Control	Outcomes
Galvao DA *et al.* 2018^12^	RCT	Exercise (aerobic and resistance) vs stretching	12-week program with supervised intervention three times per week. Resistance exercise comprised 10–12 RM, which was increased at a rate of 5–10% for the next session. Aerobic exercise comprised 2–30 mins at 60–85% of maximal heart rate	*N*=28 Age=69.7±7.6 BMI=28.9±4.1 ADT= 27 (96.4)	*N*=29 Age=70.4±9.3 BMI=28.5±4.0 ADT=27 (93.1)	Fatigue, 6 m walk test, 400 m walk test, and up and go time
Newton RU *et al.* 2020^13^	RCT	Aerobic and resistance vs stretching	Resistance training was done at an intensity of 6–12 RM for 2–4 sets per exercise. The aerobic component consisted of jogging/walking on a treadmill, and cycling or rowing at an intensity of 60–85% estimated maximum heart rate for 25–40 min	*N*=54 Age=69.0±6.3 BMI= 27.5±4.4	*N*=50 Age=67.5±7.7 BMI=28.3±3.9	6 m walk test and 400 m walk test
Piraux E *et al.* 2020^14^	RCT	Resistance training vs usual care	Resistance training comprised of 1–3 sets of 8–12 repetitions using resistance bands or dumbbells for about 30 min	N= 24 Age= 67.9±7.1 BMI=26.1±2.9	N= 24 Age= 71.9±8.1 BMI= 25.8±4.4	Fatigue and QoL
Cormie P *et al.* 2013^15^	RCT	Resistance training vs usual care	Twice weekly sessions for 12 weeks of 60 mins each with 5 min warmup and 110 min cool down period consisting of low aerobic exercise and stretching	*N*=10 Age=73.1±7.5 BMI=29.1±3.1 Time for diagnosis of bone metastasis (years)= 1.0±1.1	*N*=10 Age= 71.2±6.9 BMI= 28.3±4.0 Time for diagnosis of bone metastasis (years)= 1.0±1.0	Physical functions, fatigue, and QoL
Ashton RE *et al.* 2021^11^	RCT	Resistance exercise vs usual care	Weekly sessions using resistance bands for 6 months. It was performed with 30–60 s interpolated rest intervals until three sets of each exercise has been performed	*N*=20 Age= 64.6±6.2 BMI= 28.1±3.5	*N*=22 Age= 66.9±6.8 BMI= 28.3±4.1	QoL and fatigue
Ndjavera W *et al.* 2020^16^	RCT	Aerobic and resistance vs usual care	Two supervised session every week for 12 weeks. Each session lasted for 60 mins and consisted of aerobic interval exercise on a cycle ergometer followed by resistance training	*N*=24 Age= 71.4±5.4 BMI= 28.4±3.1	*N*=26 Age= 72.5±4.2 BMI= 27.7±3.4	QoL and fatigue
Winters-stone KM *et al.* 2014^17^	RCT	Intensity resistance training vs stretching	Two supervised sessions and one home-based session every week for 12 months	*N*=29 Age= 69.9±59.3 BMI= 28.4±4.1	*N*=22 Age= 70.5±7.8 BMI= 29.6±4.8	QoL and fatigue
Taaffe DR *et al.* 2017^18^	RCT	Aerobic and resistance training vs usual care	Resistance training comprised of 2–4 sets of each exercise at an intensity of 6–12 RM. The aerobic component comprised of 20–30 mins of exercise at 60–75% of maximal heart rate.	*N*=54 Age= 69±9.3 Time for diagnosis of bone metastasis (months)= 5.3±7.6	*N*=48 Age= 68.4±9.1 Time for diagnosis of bone metastasis (months)= 3.7±3.7	Fatigue
Langlais CS *et al.* 2023^19^	RCT	Aerobic and resistance training vs usual care	Resistance exercise consisted of three sessions per week for 12 weeks and sets progressed from 1 to 4 of 4–14 repetitions. Aerobic sessions occurred three times a week using a cycle ergometer	Aerobic *N*=8 Resistance *N*=7	*N*=10	Fatigue and QoL
Segal RJ *et al.* 2003^20^	RCT	Resistance training vs control	Supervised resistance sessions were carried out three times a week for 12 weeks at 60–70% of 1-RM.	*N*= 82 Age= 68.2±7.9 BMI= 29.0±3.5	*N*=73 Age= 67.7±7.5 BMI= 28.5±3.7	Fatigue and QoL
Reed SNC *et al.* 2009^21^	RCT	Aerobic and light resistance vs wait-list	Sixteen weeks of aerobic and light resistance training.	*N*=53 Age= 67.2±8.8	*N*=47 Age= 68.0±8.0	Fatigue and QoL
Segal RJ *et al.* 2009^22^	RCT	Aerobic, Resistance, and Control	Resistance training comprised of three sessions per week with two sets of 8–12 repetitions. Aerobic training participants exercised thrice weekly on a cycle ergometer, treadmill, or elliptical trainer beginning at 50% to 60% and later increasing to 70–75% of their predetermined peak oxygen consumption. The exercise program lasted for 24 weeks	Aerobic *N*=40 Age= 66.2±6.8 BMI= 28.9±3.4 Resistance *N*=40 Age= 66.4±7.6 BMI= 28.1±3.5	*N*=41 Age= 65.3±7.6 BMI= 29.0±4.2	Fatigue and QoL
Galvao DA *et al.*, 2014^29^	RCT	Resistance and aerobic training vs physical activity	Progressive resistance and aerobic training twice per week for 6 months. The resistance exercises progressed in loading from 12- to 6-repetition maximum (RM) for two to four sets per exercise. The aerobic training included 20–30 min of cardiovascular exercises at 70–85% maximum heart rate and perceived exertion at 11–13.	*N*=50 Age= 71.9 (5.6) BMI=24.9 (3.3)	*N*=50 Age=71.5 (7.2) BMI= 24.9 (3.1)	400 m walk test
Nilsen *et al.*, 2015^7^	RCT	High-load strength training vs usual care	Three sessions/week for 16 weeks. The first two weeks included low resistance at 40–50% of one RM, the rest of the training programme followed a linear progression in training volume from one to three sets of 10 RM on Mondays, and from two to three sets of six RM on Fridays	*N*=28 Age= 66(6.6) ADT=17 (8.7)	*N*=30 Age= 66 (5) ADT= 18 (8.2)	QoL and fatigue
Buffart *et al.*, 2015^28^	RCT	Aerobic and resistance exercise vs physical activity	The resistance exercises progressed from 12 RM to 6 RM for–4 sets per exercise. Aerobic exercises were included for 20–30 min at 70% to 85% of the maximum heart rate. An additional 90 min of home-based aerobic exercise per week was advised to complement the 60 min of supervised aerobic exercise training using the same intensity as prescribed during supervised exercise sessions	*N*=50 Age=71.9 (5.6) BMI= 24.9 (3.3)	*N*=50 Age=71.5 (7.2) BMI= 24.9 (3.1)	QoL
Sheill *et al.* 2023^23^	Multicentre two-armed randomised controlled trial (RCT)	Aerobic exercise vs standard care	The exercise group participated in a 6-month moderate to vigorous intensity aerobic exercise comprising a weekly class and a home-based aerobic exercise programme. Whereas the control group was offered the standard physical activity	*N*=30 Age (years)= 69.8±7.0 BMI (kg/m^2^)=28.4±4.84 Time since cancer diagnosis (months)= 37.36 (32.30)	*N*=31 Age (years)= 69.9±7.5 BMI (kg/m^2^)=29.9±4.35 Time since cancer diagnosis (months)=30.23	QoL
Cormie P *et al.* 2014^24^	RCT	Aerobic and Resistance training vs usual care	The exercise intervention involved twice weekly exercise sessions for 3 months. The sessions were ~60 min in duration. The aerobic exercise component included 20–30 min of cardiovascular exercise and target intensity was set at ~70–85% of the estimated maximum heart rate. The resistance training intensity was manipulated fa rom 6–12 repetition maximum and was increased by a 5–10% increment for the next set/training session	*N*=32 Age, years= 69.6±6.5 BMI, kg/m^2^= 29.3±4.5 Time since ADT injection, days=6.2±1.6	*N*=31 Age, years= 67.1±7.5 BMI, kg/m^2^= 29.6±2.6 Time since ADT injection, days=5.6±2.0	Fatigue, 6 m walk test and 400 m walk test
Windsor *et al.*, 2004^25^	RCT	Aerobic training vs usual care	Home-based, moderate-intensity, continuous walking for 30 min on at least 3 days of each week of radiotherapy at a target heart rate of 60–70% calculated maximum heart rate (as a guide to the intensity of the activity)	*N*=32 Age (years)=68.3±0.9 Weight=81.6±2.57	*N*=33 Age (years)= 69.3±1.3 Weight (kg)=82.9±1.76	Fatigue
Bourke L *et al.*, 2018^26^	RCT	Aerobic exercise vs usual care	Aerobic ET was undertaken for 12 months with intensity set at between 65 and 85% of age-predicted maximum heart rate or 12 to 17 on the Borg rating of perceived exertion (RPE) scale	*N*=25 Age (years)=68 (6) BMI (Kg.m^2^) =26.7 (2.4)	*N*=25 Age (years)=67 (9) BMI (Kg.m^2^)= 27.7 (3.2)	QoL
Monga U *et al.*, 2007^27^	RCT	Aerobic training vs patient education	The exercise protocol consisted of a 10 min warm-up, a 30 minaerobic segment consisting of walking on a treadmill, and a 510 min cool-down period	*N*=11 Age (years)= 68+4.2 Weight (lb)= 177.3+29.1 PSA 7.4+5.7	*N*=10 Age= 70.6+5.3 Weight (lb)= 80.1+28.8 Mean PSA= 6.4+5.0	Fatigue (PFS)

6MWT, 6 min walk test; FACT-P, functional assessment of cancer therapy-prostate; PFS, piper fatigue scale; RM, repetition maximum.

**Table 2:** Outcomes.

Author (year)	Fatigue	Quality of life	Physical functions
Galvao DA *et al.* 2018^12^	No change in fatigue (*P*=0.964) was assessed by the functional assessment of chronic illness therapy	**—**	6 m usual walk Exercise Baseline:4.5±0.9 12 weeks/3 month:4.8±1.0 Control Baseline:4.6±1.1 12 weeks/3 month:4.6±1.3 400 m walk Exercise Baseline: 249.1±38.7 12 weeks/3 month:245.2±32.9 Control Baseline: 252.0±47.7 12 weeks/3 month: 249.3±41.0 Up and go Exercise Baseline: 7.5±2.4 12 weeks/3 month:7.5±2.5 Control Baseline: 6.9±1.6 12 weeks/3 month:6.8±1.4
Newton RU *et al.* 2020^13^	**—**	**—**	6 m usual walk Exercise Baseline: 4.2±0.6 6 months: 4.4±0.6 Control Baseline: 4.2±0.7 6 month: 4.4±0.5 400 m walk Exercise Baseline: 246.8±36.1 6 months: 241.9±37.8 Control Baseline: 258.7±53.8 6 months: 263.7±56.9
Piraux E *et al.* 2020^14^	CTRF measured using FACIT-F Exercise Baseline: 41.2±7.7 End: 40.5±9.8 Control Baseline: 41.1±9.0 End: 35.3±12.1	Cancer-related QoL using FACT-G. Exercise Baseline: 83.5 (95% CI=75.5–91.8) End: 82.5 (95% CI=72.3–93.9) Control Baseline: 79.3 (95% CI=73.3–83.3) End: 77.9 (95% CI=67.5–85.4)	
Cormie P *et al.* 2013^15^	MFSI-SF Exercise Baseline: 5.2±16.8 12weeks: 8.8±24.9 Control Baseline: 6.0±12.3 12weeks: 3.8±13.7	SF-36 Exercise Baseline: 44.2±9.0 12weeks: 46.5±9.4 Control Baseline: 45.0±11.4 12weeks: 45.8±7.8	6 m usual walk Exercise Baseline: 4.48±0.54 12weeks: 4.23±0.33 Control Baseline: 4.45±0.56 12weeks: 4.76±0.42 400 m walk Exercise Baseline: 252.1±40.8 12weeks: 246.9±32.9 Control Baseline: 280.8±53.0 12weeks: 286.5±50.5 Timed Up and go Exercise Baseline: 7.41±1.50 12 weeks: 6.97±1.02 Control Baseline: 7.59±1.91 12weeks: 7.32±1.17
Ashton RE *et al.* 2021^11^	Brief Fatigue Inventory (BFI) Exercise Baseline: 1.2±1.2 Control Baseline: 1.9±1.4 Mean difference at 6 months: −0.1 (−0.9, 0.6)	FACT-G Exercise Baseline: 91.9±10.0 Control Baseline: 88.8±12.1 Mean difference at 6 months: 0.9 (−4.0, 5.7)	**—**
Ndjavera W *et al.* 2020^16^	FACIT-F Exercise Baseline: 41.8±10.2 6 months: 43.7±8.6 Control Baseline: 42.9±8.4 6 months: 39.9±9.3	FACT-P Baseline: 119±19 6 months: 126±15 Control Baseline: 123±16 6 months: 120±16	**—**
Winters-stone KM *et al.* 2014^17^	Schwartz Cancer Fatigue Scale Baseline: 9.87±4.47 12 months: 8.83±3.19 Control Baseline: 9.92±3.58 12 months: 9.83±3.66	QLQC30 Baseline: 87.5±14.3 12 months: 93.3±9.0 Control Baseline: 89.7±15.3 12 months: 86.7±20.7	**—**
Taaffe DR *et al.* 2017^18^	QLQC30 Exercise Baseline: 23.4±18.1 6 months: 21.9±18.4 Control Baseline: 25.8±20.2 12 months: 24.6±17.7		**—**
Langlais CS *et al.* 2023^19^	Control Baseline: 24.4±20.2 12 weeks: −2.2 (12.6) Mean change (SD) Resistance Baseline: 16.7±8.3 12 weeks: 0.0 (7.2) Mean change (SD) 12 weeks vs control (95% CI): 2.2 (−16.3–20.8) Aerobic Baseline: 36.1±27.1 12 weeks: 4.4 (6.1) Mean change (SD) 12 weeks vs control (95% CI): 6.7 (−3.8–17.1)	Control Baseline: 93.9±9.9 12 weeks: −6.0 (8.6) Mean change (SD) Resistance Baseline: 96.7±5.6 12 weeks: −1.1 (5.0) Mean change (SD) 12weeks vs control (95% CI): 4.9 (−2.4–12.2) Aerobic Baseline: 91.7±5.9 12weeks: −1.3 (9.9) Mean change (SD) 12 weeks vs control (95% CI): 4.7 (−7.6–16.9)	
Segal RJ *et al.* 2003^20^	Exercise Baseline: 40.8±10.6 12 weeks: 41.6±10.5 Control Baseline: 42.5±8.5 12 weeks: 40.3±9.4	FACT-P Exercise Baseline: 118.2±16.7 12 weeks:120.2±15.9 Control Baseline: 120.9±13.6 12 weeks: 117.6±14.9	**—**
Reed SNC *et al.* 2009^21^	FSS Exercise (*N*=37) Baseline: 4.49±1.45 16 weeks: 4.15±1.58 Control (*N*=24) Baseline: 4.50±1.33 16 weeks: 4.46±1.12	EORTC-30 Exercise (*N*=40) Baseline: 70.42±17.39 16 weeks: 73.12±15.96 Control (*N*=25) Baseline: 71.33±18.65 16 weeks: 69.00±15.12	**—**
Segal RJ *et al.* 2009^22^	FACT-F Control Baseline: 44.6±8.7 24 weeks: 42.1±8.8 Resistance Baseline: 42.8±8.7 24 weeks: 45.1±9.1 Aerobic Baseline: 44.1±8.7 24weeks: 44.2±8.9	FACT-P Control Baseline: 37.1±6.4 24weeks: 36.0±6.4 Resistance Baseline: 37.4±6.4 24weeks: 37.7±6.7 Aerobic Baseline: 37.5±6.4 24weeks: 37.8±6.5	**—**
Galvao DA *et al.*, 2014^29^		EORTC QLQ-C30 Exercise Baseline: 77.3±16.7 12 months: 76.9±16.0 Control Baseline: 78.5±15.9 12 months: 75.0±17.8	
Nilsen *et al.*, 2015^7^	EORTC QLQ-C30 symptom scales Exercise: *N*=28 Baseline: 34.5±15.2 16 weeks: 33.7±16.1 Control: *N*=30 Baseline: 36.5±14.9 16 weeks: 33±22.3	EORTC QLQ-C30 Exercise: *N*=27 Baseline: 76.5±17.3 16 weeks: 79.6±17 Control: *N*=30 Baseline: 66.7±19.6 16 weeks: 78.9±20.7	
Buffart *et al.*, 2015^28^			400 m Test Exercise: Baseline: 288.0±7.6 12 months: 270.4±7.3 Control: Baseline: 276.5±7.6 12 months: 270.4±7.3
Sheill *et al.* 2023^23^	—	Exercise: Baseline=120.3+21.096 6 months= 120.89+24.674 Control: Baseline=119.96+20.733 6 months=125.12+21.525	
Cormie P *et al.* 2014^24^	Fatigue (FACIT-Fatigue): Exercise Baseline=43.7 (8.3) 3-months=43.8 (6.8) Control Baseline= 44.8 (8.5) 3-months= 41.4 (9.5)	—	400 m walk, s: Exercise group: Baseline=260.9 (44.3) 3-months= 254.4 (42.8) Control group: Baseline= 248.5 (36.8) 3-months=253.0 (40.6) 6 m walk – usual pace, s: Exercise group: Baseline= 4.36 (0.65) 3-months=4.32 (0.62) Control group: Baseline=4.04 (0.57) 6-months=4.20 (0.38)
Windsor *et al.*, 2004^25^	Men in the control group had significant increases in fatigue scores from baseline to the end of radiotherapy (*P*=0.013), with no significant increases observed in the exercise group (*P*=0.203).	—	—
Bourke L *et al.*, 2018^26^	—	Intervention group: Baseline=71 (64, 79) 12-month=84 (80, 87) Control group: Baseline=71 (64, 78) 12-month=79 (73, 85)	—
Monga U *et al.*, 2007^27^	Intervention group: Preradiotherapy= 2.4+2.4 postradiotherapy= 0.8+1.8 Difference=−1.6+2.0 Control Group: Preradiotherapy= 1.1+1.9 Postradiotherapy= 3.8+2.2 Difference=2.7+2.2	—	—

CTRF, cancer treatment-related fatigue; EORTC QLQ-C30, European organization for research and treatment of cancer quality of life questionnaire C30; FACIT-F, functional assessment of chronic illness therapy-fatigue; FACT-F, functional assessment of cancer therapy-fatigue; FACT-G, functional assessment of chronic illness therapy-general; FACT-P, functional assessment of cancer therapy-prostate; FSS, fatigue subscale; MFSI-SF, multidimensional fatigue symptom inventory short form; QLQC30, quality of life questionnaire.

### Risk of bias

Most of our studies were low-risk in six of the seven components of the Cochrane ROB tool. One exception was the component of blinding of participants/personnel, in which 15/20 studies were marked as high-risk, while two studies were categorized as low-risk and three as unclear. The detailed risk of bias is presented in Table 3.

**Table 3:** Risk of bias.

Author (year)	Random sequence generation	Allocation concealment	Selective reporting	Blinding of participants/personnel	Blinding of outcome assessment	Incomplete outcome data	Other sources of bias
Galvao DA *et al.* 2018^12^	Low risk	Low risk	Low risk	High risk	Unclear	Low risk	Low risk
Newton RU *et al.* 2020^15^	Low risk	Low risk	Low risk	High risk	Unclear	Low risk	Low risk
Piraux E *et al.* 2020^16^	Low risk	Low risk	Low risk	High risk	Unclear	Low risk	Low risk
Cormie P *et al.* 2013^17^	Low risk	Low risk	Low risk	High risk	Low risk	Low risk	Low risk
Ashton RE *et al.* 2021^13^	Low risk	Low risk	Low risk	High risk	Unclear	Low risk	Low risk
Ndjavera W *et al.* 2020^18^	Low risk	Low risk	Low risk	High risk	Low risk	Low risk	Low risk
Winters-stone KM *et al.* 2014^19^	Low risk	Unclear	Low risk	High risk	Unclear	Low risk	Low risk
Taafe DR *et al.* 2017	Low risk	Low risk	Low risk	High risk	Unclear	Low risk	Low risk
Langlais CS *et al.* 2023^21^	Low risk	Unclear	Low risk	High risk	Low risk	Low risk	Low risk
Segal RJ *et al.* 2003^22^	Low risk	Low risk	Low risk	High risk	Unclear	Unclear	Low risk
Reed SNC *et al.* 2009	Low risk	High risk	Low risk	High risk	Low risk	Low risk	Low risk
Sejal RJ *et al.* 2009	Low risk	Low risk	Low risk	High risk	Unclear	Low risk	Low risk
Buffart *et al.*, 2015^29^	Low risk	Low risk	Low risk	High risk	High risk	Low risk	Low risk
Nilsen *et al.*, 2015^30^	Low risk	Low risk	Low risk	Unclear	Unclear	Low risk	Low risk
Galvao DA *et al.*, 2014	Low risk	Low risk	Low risk	Unclear	Unclear	Low risk	Low risk
Sheill *et al.* 2023^25^	Low risk	Low risk	Low risk	Low risk	Low risk	Low risk	Low risk
Cormie P *et al.* 2014^26^	Low risk	Low risk	Low risk	High risk	Low risk	Low risk	Low risk
Windsor *et al.*, 2004^27^	Unclear	Unclear	Low risk	Low risk	Low risk	Low risk	Low risk
Bourke L *et al.*, 2018^28^	Low risk	Low risk	Low risk	Unclear	Low risk	Low risk	Low risk
Monga U *et al.*, 2007^7^	Unclear	Unclear	Low risk	High-risk	Low risk	Low risk	High-risk

## Results of the meta-analysis

### QoL

**Figure 2:**
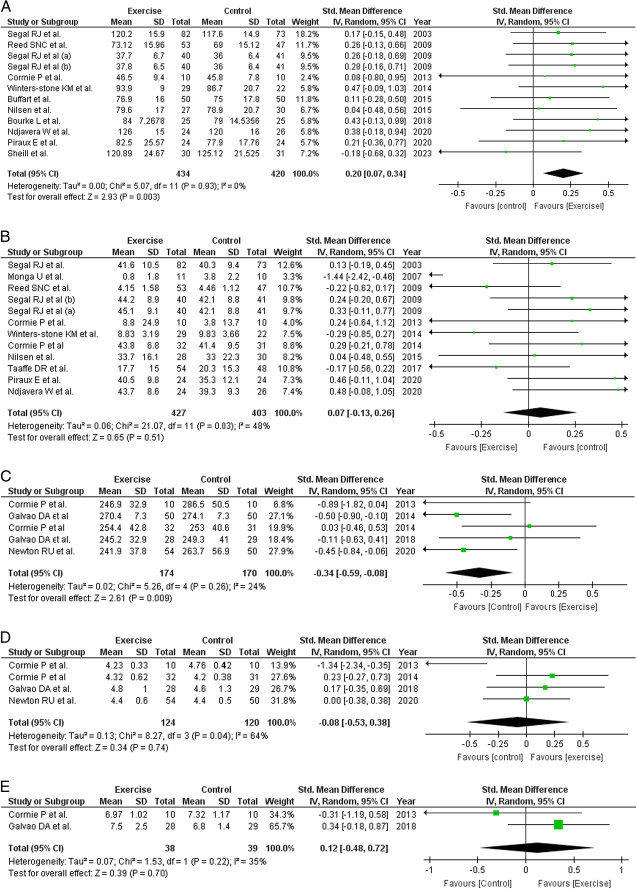
Forest Plots. (A) QoL (B) Fatigue (C) 400-meter walk test (D) 6-meter walk test (E) Up and go time.

Of the 20 studies included in this review, 13 reported this outcome. There were 854 patients in the exercise group, consisting of 434 patients whereas there were 420 patients assigned to the control group. However, two studies could not be included in the analysis^13,21^ because of the lack of data needed for the analysis. The pooled results revealed a statistically significant association between the exercise group compared to the control group in improving the QoL of the patients (SMD= 0.20, 95% CI=0.07–0.34, *P*=0.003, *I* ^2^=0%) (Fig. 2).

### Fatigue

This outcome was reported in 15 studies. However, four studies^13,14,21,27^ could not be included in the final analysis because of a lack of relevant data. Of the 830 patients included in this study, 427 were in the intervention group and 403 were in the control group. A nonsignificant effect of exercise on fatigue was observed compared to the control (SMD=0.07, 95%CI=−0.13, 0.26, *P*=0.51, *I* ^2^=49%). Similarly, sensitivity analysis was performed by removing the study by Monga *et al*. However, no statistically significant differences were observed between the groups (Fig. 2).

### 400 m walk test

Our third outcome, a 400 m walk test, was reported in only five of the recruited articles. In total, 344 patients with 174 assigned to the exercise and control groups. Surprisingly, the pooled results significantly favored the control group for the 400 m walk test compared to the interventional group (Fig. 2).

### 6 meter walk test

This outcome was reported in four of the 20 recruited studies. In total, 244 patients with 124 assigned to the exercise and control groups. Our analysis showed no significant association between exercise and the 6 m walk test when compared to the control group (SMD=−0.08, 95%CI=−0.53−0.38, *P*=0.74, *I* ^2^=64%). Similarly, the sensitivity analysis showed no benefit (Fig. 2).

### Up-and-go time

This outcome was reported in only two studies, with a total of 77 patients. The exercise and control groups included 38 and 39 patients, respectively. Our analysis showed no significant association between exercise and up-and-go time when compared with the control group (SMD= 0.12, 95% CI=−0.48–0.72, *P*=0.70, *I* ^2^=35%) (Fig. 2).

#### Publication bias

**Figure 3:**
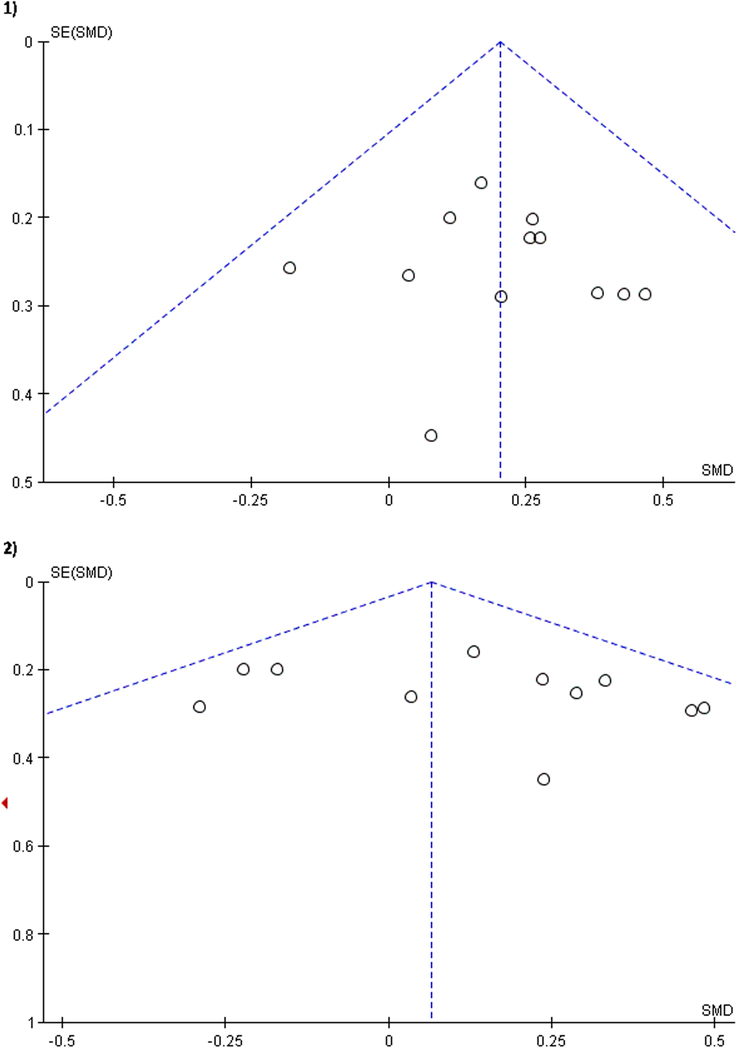
Funnel plots 1) Fatigue 2) QoL.

The publication bias was assessed using a funnel plot (Fig. 3). The plots showed some degree of asymmetry, which may be reflective of reporting bias. Consequently, it is difficult to estimate the effect size.

## Discussion

This study aimed to evaluate the association between exercise and its effects in patients with PCa. Our study results established a significant association between exercise and overall elevation in the QoL (*P*=0.003), which is in line with the study by Bourke *et al*.^28^, where a significant relationship was quantified between exercise and cancer-specific (QoL). In contrast to previous studies, the most recent meta-analysis yielded a different result, indicating an association that favored exercise; however, it was statistically insignificant (*P*=0.718)^32^. This could be explained by the fact that our analysis did not include any overlapping interventions (dietary, psychological, etc.), which may have led to different results, indicating that exercise alone is far superior to mixed interventions for these demographics.

However, our analysis showed no positive interaction between exercise and fatigue (*P*=0.51), which contradicts the results of previous studies. Anderson *et al*. reported a relationship between the two, similar to Lopez *et al*., who showed that exercise has significant positive benefits in patients with PCa^32,33^. This might be attributed to differences in the inclusion criteria of the studies. Anderson *et al*. included studies with mixed interventions (exercise with dietary changes), whereas Lopez *et al*. only considered resistance training. However, our results strengthen the conclusion of Kelley *et al*.^34^, who pointed out the ambiguity in research across exercise and its effect on fatigue in their grand review, demonstrating that caution should be taken when establishing a relationship between them. The lack of a significant association between exercise and fatigue is an intriguing finding that warrants further investigation. Fatigue reduction is an important aspect of QoL improvement. Fatigue can profoundly impact daily functioning, emotional well-being, and the overall QoL. Therefore, it is reasonable to expect that exercise interventions aimed at improving QoL will lead to a reduction in fatigue levels. However, the results of this meta-analysis contradict those of other studies. This may be because of the specific exercise interventions employed in these studies. Different exercise modalities, intensities, and durations may have varying effects on fatigue levels. It should be noted that we did not include Languis *et al*. and Ashton *et al*. due to a lack of clarity of data, but overall, both demonstrated modest effects on the intervention on QoL and fatigue, respectively^13,21^.

Another factor to consider is the potential impact of the various treatment techniques on fatigue. Patients with PCa are frequently subjected to various therapies including surgery, radiation therapy, and chemotherapy, all of which can lead to fatigue. The presence of treatment-related weariness may have attenuated the benefits of exercise on fatigue, as observed in this meta-analysis. Furthermore, the association between exercise and exhaustion might be modified by individual patient features as well as the participants’ general health status. Patients with PCa can have a wide range of characteristics including differences in disease stage, comorbidities, and general fitness levels.

While the lack of a link between exercise and fatigue is notable, it is necessary to recognize that improving QOL involves a greater scope than fatigue reduction. Even if the specific symptoms of fatigue do not improve significantly, exercise therapies may have broader effects on psychological well-being, social relationships, and the overall QOL. Because of the significant relationship found between exercise and QOL in this meta-analysis, future research should focus on optimizing exercise interventions, investigating the potential synergistic effects of exercise with other treatment modalities, and identifying specific patient characteristics that may influence the exercise response.

Other outcomes of interest were the 400 m walk test, the 6 m walk test, and the up-and-go time, all of which were found to have no significant association with our intervention. The control group showed a significant improvement in the 400 m walk test. It is crucial to note that the number of studies reporting this outcome was minimal, which may limit the generalizability of the findings. Further research is required to explore the potential reasons for this counterintuitive finding. Our results are contrary to those of another meta-analysis that reported a significant improvement in cardiovascular fitness^32^. This could be explained by the fact that our study only evaluated cardiovascular fitness via walking tests, while previous studies assessed cardiovascular fitness with VO2 max combined with other modalities such as the 600 m walk test, leading to mismatched results with ours.

It is important to consider that physical functioning outcomes are modifiable and, thus, can be influenced by various factors beyond exercise interventions alone. Patient characteristics, disease progression, and other interventions or treatments may interact with exercise interventions and impact observed outcomes. Additionally, the specific types and intensities of exercise interventions used in the included studies may have contributed to the lack of significant effects on physical functioning measures. It is imperative to mention that there are multiple exercises, such as flexibility and stretching exercises, balance and coordination exercises, mind-body practices, interval training, and functional training, all of which may reproduce different results.

While the lack of significant associations between exercise and the 400 m walk test, the 6 m walk test, and the up-and-go time are intriguing, it is crucial to interpret these findings within the context of the available literature and the limitations of the included studies. Future research should explore alternative exercise interventions, consider the influence of other factors on physical functioning outcomes, and increase the number of studies reporting on these measures to provide more robust evidence.

Several limitations of the studies included in this meta-analysis should be noted. First, the studies included in this meta-analysis had high heterogeneity in terms of participant characteristics such as age, disease stage, treatment history, and overall health status. These variations can introduce confounding factors that may have influenced the outcomes and contributed to the inconsistencies observed across studies. Furthermore, the outcome assessment tools and specific exercise interventions used in the included studies varied significantly in terms of type, intensity, duration, and frequency. These variations can lead to differences in the effectiveness of the interventions, making it challenging to draw definitive conclusions regarding the effects of exercise on the outcomes of interest. Additionally, the sample sizes of individual studies were relatively small, which affected the statistical power to detect significant associations. Moreover, the limited number of studies reporting certain outcomes, such as the 400 m walk test, the 6 m walk test, and the up-and-go time, further restrict the generalizability and reliability of the findings for these specific measures. The small number of studies reporting these outcomes reduced the overall sample size and may limit the representativeness of the results. Lastly, an important consideration is potential publication bias, where studies with positive or significant results are more likely to be published, whereas studies with null or negative results may be under-represented. This bias can skew the overall findings and lead to an overestimation of the true effect sizes.

## Conclusion

This review highlights the critical role of exercise in improving the QoL of patients with PCa. These findings have broad implications for health care practices and policies. Clinically, integrating exercise into cancer rehabilitation programs is paramount and requires healthcare professionals to prescribe and oversee personalized exercise plans. A collaborative approach involving oncologists, physical therapists, and exercise physiologists is essential for safe and effective implementation of exercise. Policymakers and healthcare systems should invest in infrastructure to facilitate physical activity for cancer patients, including dedicated exercise spaces and community programmes. This study also acts as a springboard for future research to explore comprehensive exercise protocols and address challenges such as fatigue and physical activity in cancer treatment. Further research should examine the role of digital health technologies in enhancing exercise adherence. Overall, these insights underscore the importance of exercise in cancer care, enhancing patient outcomes, and overall quality of life.

## Ethical approval

This is systematic review and metanalysis PROSPERO 2023 CRD42023432859. Available from: https://www.crd.york.ac.uk/prospero/#recordDetails.

## Consent

Not applicable.

## Sources of funding

The authors declare that no funds, grants, or other support was received during the preparation of this manuscript.

## Author contribution

S.N., A.M., F.A., W.F., and W.H.: conceptualization; S.A., M.J., A.A., M.A., S.F., M.F.O., K.R.V., and I.C.O.: data curation; O.A.E., M.G., S.A., M.J., A.A., M.A., and S.F.: formal analysis; S.A., M.J., A.A., M.A., S.F., M.F.O., K.R.V., and I.C.O.: investigation; O.A.E., M.G., S.A., M.J., A.A., M.A., and S.F.: methodology; A.M., S.N., F.A., W.F., and W.H.: project administration; M.A., S.A., M.J., and S.F.: resources; S.F., M.F.O., K.R.V., and I.C.O.: software; W.H. and I.C.O.: supervision; M.J., S.F., M.F.O., and K.R.V.: validation; W.H., A.R., O.A.E., M.G., S.A., M.J., A.A., M.A., S.F., and M.F.O.: visualization; S.N., A.M., F.A., W.F., and W.H.: writing – original draft; S.N., A.M., F.A., W.F., W.H., A.R., O.A.E., M.G., S.A., M.J., A.A., M.A., S.F., M.F.O., K.R.V., and I.C.O.: writing – review and editing.

## Conflicts of interests disclosure

The authors have no relevant financial or non-financial interests to disclose.

## Research registration unique identification number (UIN)

PROSPERO 2023 CRD42023432859 Available from: https://www.crd.york.ac.uk/prospero/#recordDetails.

## Guarantor

Iván Cherrez-Ojeda and Wael Hafez.

## Data availability statement

The data supporting the findings of this study are available from the corresponding author and first author upon request.

## Provenance and peer review

Not commissioned, externally peer-reviewed.
